# Chronic Pain in the ICD-11: New Diagnoses That Clinical Psychologists Should Know About

**DOI:** 10.32872/cpe.9933

**Published:** 2022-12-15

**Authors:** Antonia Barke, Beatrice Korwisi, Winfried Rief

**Affiliations:** 1 Clinical Psychology and Psychological Intervention, Institute for Psychology, University Duisburg-Essen, Essen, Germany; 2 Department of Psychology, Clinical Psychology and Psychotherapy, Philipps University of Marburg, Marburg, Germany; University of Zurich, Zurich, Switzerland

**Keywords:** ICD-11, classification, biopsychosocial model of chronic pain, chronic primary pain, chronic secondary pain, implementation

## Abstract

**Background:**

In the 10th revision of the International Classification of Diseases and Related Health Problems (ICD-10), chronic pain was not represented adequately. Pain was left undefined and not recognized as a biopsychosocial phenomenon. Instead, a flawed dualism between psychological and somatic factors was implied. Individual diagnoses were ill-defined and scattered randomly through different chapters. Many patients received diagnoses in remainder categories devoid of meaningful clinical information.

**Method:**

The International Association for the Study of Pain launched a Task Force to improve the diagnoses for the 11th revision of the ICD and this international expert team worked from 2013-2019 in cooperation with the WHO to develop a consensus based on available evidence and to improve the diagnoses.

**Results:**

A new chapter on chronic pain was created with a biopsychosocial definition of pain. Chronic pain was operationalized as pain that persists or recurs longer than three months and subdivided into seven categories: Chronic primary pain and six types of chronic secondary pain. All diagnoses were based on explicit operationalized criteria. Optional extension codes allow coding pain-related parameters and the presence of psychosocial aspects together with each pain diagnosis.

**Conclusion:**

First empirical studies demonstrated the integrity of the categories, the reliability, clinical utility, international applicability and superiority over the ICD-10. To improve reliability and ease of diagnosis, a classification algorithm is available. Clinical psychologists and other clinicians working with people with chronic pain should watch the national implementation strategies and advocate for multimodal and interdisciplinary treatments and adequate reimbursement for all providers involved.

## Highlights

- A systematic chapter on chronic pain in the ICD-11 improves the representation of chronic pain.
- Chronic pain is pain that persists or recurs for more than three months.
- Chronic pain in the ICD-11 is regarded as biopsychosocial.
- ICD-11 introduces chronic primary pain and distinguishes six types of chronic secondary pain.

## Background

### What Was Wrong With the Representation of Chronic Pain in the ICD-10?

In the previous version of the International Classification of Diseases and Related Health Problems (ICD), the ICD-10, chronic pain was represented neither systematically nor adequately. The main shortcomings were: Firstly, the ICD-10 did not reflect the widely accepted biopsychosocial model of pain (Rief et al., 2010; Rief et al., 2008; Treede et al., 2010), which is also a central aspect of the internationally widely accepted definition of pain by the *International Association for the Study of Pain* (IASP) (Raja et al., 2020). Secondly, for many important types of chronic pain, no diagnoses were available at all: Chronic neuropathic pain, chronic pain associated with cancer and its treatment, or chronic pain after surgery or accidents were missing in the ICD-10 (Rief et al., 2012). Thirdly, even if a diagnosis was available in the ICD-10, it often lacked clear definitions and criteria, e.g., “R52.2 Other chronic pain”. In most cases, not even the information whether the pain was chronic or acute could be recorded (e.g., “M54.4 Low back pain”) – despite agreement that highly relevant differences exist between acute and chronic pain (Kröner-Herwig, 2017; Treede, 2019). As a result, one of the most frequently used diagnoses for chronic pain was the ill-defined residual category “R52.2 other chronic pain”, which held next to no information value for clinicians, patients or health statistics. Fourthly, the diagnoses that were available in ICD-10 were scattered rather arbitrarily among different chapters (Rief et al., 2010; Rief et al., 2012), depending upon the medical specialty that tended to treat them. For example, the diagnosis “M54.5 low back pain” was found in the chapter for diseases of the musculoskeletal system and connective tissue while different types of headache (“G43 migraine”) were listed among the diseases of the nervous system (World Health Organization, 2019).

Clinical psychologists are probably most familiar with the chronic pain diagnosis “F45.4 persistent somatoform pain disorder” available in the so-called “ICD-10 F-chapter” for mental and behavioral disorders. This diagnosis recognizes the role of psychological factors in the development and maintenance of the chronic pain and gives a definition that specifies the chronic course of the pain (World Health Organization, 2019). However, the contribution of biological or physiological factors is excluded. By definition, the diagnosis F45.4 cannot be assigned if a patient has chronic pain associated with an underlying disease such as, for example, rheumatoid arthritis. This contributes to the artificial and problematic dichotomy of “psychological” vs. “somatic” chronic pain in the ICD-10 (Arnold et al., 2017; Rief et al., 2008; Treede et al., 2010). The German modification of the ICD-10 includes an additional chronic pain diagnosis, “F45.41 chronic pain with somatic and psychological factors” which, for the first time, recognized the contribution of *both* biological and psychological factors to chronic pain (Nilges & Rief, 2010) thereby overcoming the dichotomy (Arnold et al., 2017; Treede et al., 2010). This was a great step forward and the frequency with which this diagnosis has since been used (Häuser et al., 2013) shows it is well-accepted – probably because it offers a much-needed way of classifying chronic pain according to the biopsychosocial model. Despite these advances, the diagnosis F45.41 had to compromise. Its location in the chapter on mental and behavioral disorders was a theoretical compromise since chronic pain is neither. The fact that the diagnosis is only available in the German modification (ICD-10-GM), is a practical compromise since it means that the diagnostic advance is geographically limited to countries that use this national version (World Health Organization, 2022b).

### What Were the Consequences of the Deficient Representation of Chronic Pain in ICD-10?

Negative consequences arose from the inadequate representation of chronic pain in the ICD-10 for patient treatment, research into chronic pain as well as health statistics and health policies. Most importantly, the distinction of “psychological” chronic pain on the one hand and “somatic” chronic pain on the other, is not useful because chronic pain is always an interplay of psychological, biological, and social factors (Raja et al., 2020; Rief et al., 2008; Treede et al., 2010). Since in many healthcare systems, ICD codes are relevant for treatment choice and treatment access (Boerma et al., 2016; Jakob, 2018a, 2018b), patients with chronic pain may be excluded from specific multimodal interdisciplinary pain treatment programs as well as from psychological treatment (Nilges & Rief, 2010; Rief et al., 2009; Rief et al., 2008), unless they also receive a diagnosis of a mental disorder, such as F45.4. On an individual level, this meant that many patients tended to receive multimodal therapies including psychological treatments at a very late stage, often only when treatment providers and patients felt they had exhausted the somatic treatments without much progress. This made it unnecessarily hard for patients to accept the biopsychosocial model and engage with psychological treatments. Individually, this may mean more distress and suffering. At a public health level, this translates into a larger societal burden of chronic pain and direct and indirect costs (Blyth et al., 2019; Blyth & Huckel Schneider, 2018).

Missing diagnoses meant that for treatment purposes, precise and appropriate codes for the chronic pain were lacking and clinicians chose various ways of expressing chronic pain diagnoses, often with recourse to entities such as “chronic intractable pain” (R52.1). This led to numerous problems in communication with patients and health providers. Considering the role of outcome expectations that have been shown for many areas (Auer et al., 2016; Di Blasi et al., 2001; Laferton et al., 2017) labeling a person’s pain as “intractable” may convey a nihilistic therapeutic attitude to clinician and patient alike. Apart from problems of treatment and management of individual cases, the lack of diagnostic codes also rendered the different types of chronic pain and the associated burden invisible from the perspective of public health policy.

The vague definitions and ambiguous diagnoses also presented difficulties for the communication between patients and healthcare providers as well as for the information exchange among healthcare professionals. On a larger scale, it impeded the formulation of fruitful research agendas. Referring to a large variety of chronic pain syndromes as “non-cancer pain” or “non-specific pain” underestimated the differences between the syndromes – while researching only into very specific syndromes glossed over the commonalities. Finally, in epidemiological and register studies based on inadequate representation, the true prevalence of chronic pain and its associated disease burden remained underestimated. Such underestimation, in turn, was likely to influence health policy decisions and funding allocation (Blyth et al., 2019; Rice et al., 2016; Treede et al., 2010).

## Method

### Developing a New Set of Chronic Pain Diagnoses for ICD-11

To remedy the situation of chronic pain in the ICD-10, the community of pain specialists had long worked together and argued for a classification better reflecting the empirical and theoretical advances. In 2012 the IASP formed an international and interdisciplinary task force and collaborated with the World Health Organization (WHO) to reform the classification of chronic pain for the next revision of the ICD. The WHO demanded consensus and evidence in order to enter diagnoses into the ICD-11 (World Health Organization, n.d.). The Task Force provided both by striving for a consensus among the professionals working with patients with chronic pain and publishing the results in a series of papers (Aziz et al., 2019; Bennett et al., 2019; Benoliel et al., 2019; Nicholas et al., 2019; Nugraha et al., 2019; Perrot et al., 2019; Scholz et al., 2019; Schug et al., 2019; Smith et al., 2019; Treede et al., 2019; Treede et al., 2015). The development was accompanied by formative evaluations (Barke et al., 2018; Barke et al., 2022) and evaluative studies (Hay et al., 2022; Korwisi, Garrido Suarez, et al., 2022; Korwisi et al., 2020; Zinboonyahgoon et al., 2021). In 2019, the World Health Assembly endorsed the ICD-11 with the new classification of chronic pain (World Health Assembly, 2019). The ICD-11 came into effect on January 1^st^, 2022 for international mortality reporting (World Health Organization, 2022a). Many countries are currently preparing the implementation of the ICD-11 within their national healthcare systems.

## Results: The New Chronic Pain Diagnoses in ICD-11 and How They Address the Problems in ICD-10

### An Improved Definition of Chronic Pain

The chronic pain classification implemented in the ICD-11 forms one structured chapter, which contains all chronic pain diagnoses in one logical order (for details see below), which are subdivisions of the clearly operationalized entity “chronic pain” (MG30, ID: http://id.who.int/icd/entity/1581976053)

The definition of chronic pain was aligned with the updated IASP diagnosis of pain (Raja et al., 2020): “Pain is an unpleasant sensory and emotional experience associated with, or resembling that associated with, actual or potential tissue damage.” It continues to specify chronic pain as “pain that persists or recurs for longer than 3 months”, providing a clear operationalization of chronic pain. The defining sentence is immediately followed by the clause regarding the typical nature of chronic pain: “Chronic pain is multifactorial: biological, psychological and social factors contribute to the pain syndrome.” This sentence expresses the biopsychosocial model for all types of chronic pain. It is open for variable weights of the respective factors in different chronic pain syndromes, but unequivocally affirms the general model for all subdiagnoses that characterize specific syndromes. Here it is important to note that in the ICD-11 the subordinate diagnoses (called “children”) inherit the characteristics of the higher-order diagnoses (called “parents”), without repeating all the features in each child diagnosis. Throughout the whole chapter of chronic pain, chronic pain is defined as explained here. With this definition, the ICD-11 addressed and remedied a major criticism leveled at the earlier editions, and now accurately reflects the widely accepted biopsychosocial model of pain.

### Adding Missing Diagnoses

The second major criticism was that for many important types of chronic pain, no diagnoses were available at all in the ICD-10. Diagnoses were missing for chronic neuropathic pain, chronic pain associated with cancer or its treatments, chronic pain after surgery and accidents, as well as many types of chronic orofacial pain. The ICD-11 classification contains systematically ordered diagnoses in these fields. Chronic pain has seven subdivisions:

MG30.0 Chronic primary pain (Nicholas et al., 2019)
MG30.1 Chronic cancer related pain (Bennett et al., 2019)
MG30.2 Chronic postsurgical or post traumatic pain (Schug et al., 2019)
MG30.3 Chronic secondary musculoskeletal pain (Perrot et al., 2019)
MG30.4 Chronic secondary visceral pain (Aziz et al., 2019)
MG30.5 Chronic neuropathic pain (Scholz et al., 2019)
MG30.6 Chronic secondary headache or orofacial pain (Benoliel et al., 2019)

The reasoning behind these subtypes and the diagnoses classified there have been explained and discussed in the dedicated papers for each subtype. Here we can only give a brief resumé – for fuller details we recommend the specific articles.

#### Chronic Primary Pain

Chronic primary pain is defined as chronic pain in one or more anatomical regions that is associated with significant emotional distress and/or significant functional disability (Nicholas et al., 2019). The diagnosis should be assigned unless the symptoms are better accounted for by another diagnosis in the section of chronic secondary pain.

The definition of the new diagnosis of chronic primary pain is formulated to be agnostic regarding the etiology of the pain syndrome and is purely descriptive. Subsuming a diagnostic term here does not commit us to the claim that no somatic factors contribute to the diagnosis. Neither does it commit us to the claim that psychosocial factors are the main contributors. This is true on the level of diagnostic entities: classifying Fibromyalgia as a type of chronic primary pain does not imply the empirical judgement that central sensitization or other somatic processes do not play a part in the Fibromyalgia syndrome. At the patient level, assigning a diagnosis of chronic primary back pain does not mean to deny that biological factors contribute to the chronic pain or to claim that psychological factors dominate. This descriptive nature is viewed as a distinct advantage. If another diagnosis accounts better for the chronic pain, one of the secondary diagnoses should be assigned, usually in combination with the respective underlying condition. Note, however that – again – this does not imply that no psychosocial factors may be present or relevant regarding the pain. The biopsychosocial model of chronic pain applies to chronic primary and chronic secondary pain in exactly the same way and thus psychosocial factors may be relevant in both instances. The difference is that:

- For chronic primary pain significant distress or functional interference (or both) are required as part of the definition.
- For chronic secondary pain a clearly defined somatic factor as expressed in another ICD-11 diagnosis is required and should be co-diagnosed.

In the section on chronic primary pain several frequent pain syndromes are classified, including chronic pain often referred to as 'functional gastrointestinal disorders', as characterized by the Rome criteria (Drossman & Hasler, 2016). See Table 1 for an overview.

**Table 1:** Overview of Chronic Primary Pain and its Subdiagnoses in the ICD-11

Chronic Primary Pain in the MMS Linearization (MG 30.0) / Subdiagnoses classified here	Foundation ID^a^
Chronic primary visceral pain (MG30.00)	679352876
Chronic primary chest pain syndrome	128474405
Chronic primary epigastric pain syndrome	1983908934
Chronic primary bladder pain syndrome	2093682836
Chronic primary pelvic pain syndrome	1663013388
Chronic primary abdominal pain syndrome	709631177
Chronic widespread pain (MG30.01)	849253504
Fibromyalgia syndrome	236601102
Chronic primary musculoskeletal pain (MG30.02)	1236923870
Chronic primary cervical pain	2014134682
Chronic primary thoracic pain	642165115
Chronic primary low back pain	1291385632
Chronic primary limb pain	413174579
Chronic primary headache or orofacial pain (MG30.03)	2104869000
Chronic migraine	1336990680
Burning mouth syndrome	618998878
Chronic primary orofacial pain	1545281608
Chronic primary temporomandibular disorder pains	975254799
Chronic tension-type headache	107534985
Complex regional pain syndrome (MG30.04)	1834504950
CRPS Type I	2067142665
CRPS Type II	1415867395

^a^To locate the entities using their foundation ID please use the ICD-11 Foundation Browser (https://icd.who.int/dev11/f/en) and paste the ID number in the search field. This is only required in case you would like to access the subdiagnoses that for technical reasons do not have an MG30 code. Further details and explanations regarding these technical aspects can be found in (Korwisi, Barke, et al., 2022).

The terms chronic “primary” and chronic “secondary” were adapted from the headache classification (Headache Classification Committee of the International Headache Society [IHS], 2018). They were chosen to express the fact that the chronic pain constitutes a health problem in its own right with high clinical priority for the patient and is not directly associated with another disease accounting for the pain. The term was preferred by the WHO and seen to have a number of advantages over other terms that might have been considered, such as “non-specific”, “functional” or “idiopathic”.

#### Chronic Secondary Pain

Chronic secondary pain is chronic pain that accompanies underlying diseases or health conditions that are coded elsewhere in the ICD. In this section, chronic pain in connection with cancer or its treatment (Bennett et al., 2019), chronic pain after surgery or accidents (Schug et al., 2019), chronic musculoskeletal pain due to underlying conditions such as rheumatoid arthritis (Perrot et al., 2019), chronic visceral pain due to persisting inflammation or mechanical causes (Aziz et al., 2019), chronic neuropathic pain (Scholz et al., 2019) and chronic secondary headache (Benoliel et al., 2019) (including medication overuse headache) can be classified. It should again be noted that these diagnoses are also children of chronic pain, and thus inherit the fundamental biopsychosocial model.

The diagnoses listed under chronic secondary pain address the criticism that many chronic pain conditions could not be diagnosed within ICD-10. A typical case is chronic cancer-related pain. Due to medical advances, many more people survive cancer (Glare et al., 2022). In a significant number of cases, the cancer survivors suffer from chronic pain, either due to the cancer itself or due to the often aggressive treatments needed. For both types of chronic pain codes were created: The former can be coded as “Chronic cancer pain” (MG30.10), the latter as “MG30.11 Chronic post cancer treatment pain” (MG30.11). For the affected person and their families, the diagnosis can mean better understanding and acknowledgement of the chronic pain and improvements in the access to multimodal and interdisciplinary care. Statistically, the chronic pain people suffer as a result of cancer and its therapies and the associated burden become visible and can be taken into account in health planning. The same is true for chronic neuropathic pain, chronic postsurgical pain and chronic pain after accidents.

### Addressing Unclear Criteria and Ambiguous Diagnoses

Other diagnoses were part of the ICD-10, but lacked clear criteria. This issue was addressed in the ICD-11 by introducing operationalized diagnostic criteria, which at all levels state criteria that are individually necessary and jointly sufficient for the respective diagnosis. On average, each diagnosis relies on 4-7 explicit criteria. Each diagnosis inherits the criteria of the diagnosis above and adds more specific criteria. In total, the diagnoses in the section on chronic pain are based on c. 200 explicit criteria.

### Better Representation of Relevant Factors and Pain Parameters

Given the centrality of the biopsychosocial model of chronic pain, it is justified to expect that biopsychosocial factors can be expressed better in the ICD-11. Indeed, there are several ways in which they can be coded alongside all chronic pain diagnoses, primary and secondary. The tools provided for this purpose are “extension codes”. With extension codes, information can be added to the categorical diagnoses. In the section of chronic pain, extension codes for “pain severity” and the “presence of psychosocial factors” allow the expression of psychosocially relevant information. A further extension code can be assigned to communicate “temporal features” of the pain (continuous, episodic or continuous with additional flare-ups).

The pain severity specifier captures three important aspects of chronic pain: its intensity (*How much does it hurt? How intense is the pain?*), the pain-related emotional distress experienced by the person (*How much does the pain distress you?*) and the pain-related interference with everyday life and functioning (*How much does the pain interfere with your daily life?*). All three aspects should be rated on a numerical rating scale from 0 – 10, or – if preferred – on a visual analogue scale by the patient (see Box 1 for the exact wording as well as a case vignette showing their application). The numeric scores can be used for individual documentation. However, they can also be converted into severity codes of “none – mild – moderate – severe”, which can be included with any chronic pain diagnosis in ICD-11, thereby providing a fuller picture of the chronic pain and how it affects the individual person.

Box 1 **Case Vignette: Paul (55 Years)**

History: Paul works as a mechanic in the automotive industry. About 22 months ago, Paul had been diagnosed with cancer of the prostate. He underwent surgery to remove the prostate. The surgery went well. After the initial shock of the diagnosis, he was glad that the surgery was over and he had very few side effects. On his doctor’s advice, he began a course of chemotherapy with Docetaxel. During the chemotherapy, he developed neuropathic pain in the hands and feet. He was told that in many cases the pain resolves a while after the last dose, but in some cases, it does not. For Paul, the pain did not remit.

Paul was on sick leave during the surgery and the subsequent recovery. Afterwards, he went back to work, only pausing for a few days for each course of chemotherapy. When the neuropathic pain developed, he found his work harder and harder. Hoping the pain would go away after the last treatment, he gritted his teeth and carried on working full hours despite the pain and the interference with his work. He is determined to continue in his present work schedule as a matter of pride. The family had bought a house a few years ago and there were a few years of mortgage payment left. Paul worries a lot about his pain and how it affects his and his family’s life. He finds it difficult to fall asleep due to the pain and the worry. He feels exhausted and overstretched and often withdraws from activities he used to like. His family-life suffers from his dejected mood and irritability. On a scale from 0-10 he rates his pain in the last week as “7” (“How strong was your chronic pain in the last week [on average]?”) and the pain-related interference as “5” (“How much did the pain interfere with your activities in the last week [on average]?”), his pain-related distress he rates as a “7” (“How much pain-related distress did you experience in the last week because of your pain [on average]?”).

**Table:** 

Diagnoses According to the ICD-11
MG30.11 Chronic post cancer treatment pain	
Associated with:	XS7G Psychosocial factors present
Has severity:	XS2E severe pain [pain intensity]
Has alternate severity 1:	XS7N severe distress [pain-related distress]
Has alternate severity 2:	XS2L moderate pain-related interference [pain-related interference]
Has causing condition:	2C82.Y Other specified malignant neoplasms of prostate
Final code: MG30.11&XS7G&XS5D&XS7C&XS5R/2C82	

*Note.* This code is optimized for machine readability and does not have to be memorized by humans – it is chosen via computer interface. However, it contains all of the above information. It could be augmented even further with information regarding the neoplasm itself (e.g. staging).

More specifically, the presence of psychosocial factors can be coded with the extension code “presence of psychosocial factors”. This code is designed to allow coding problematic cognitive (e.g., catastrophizing, excessive worry, Eccleston & Crombez, 2007; Sullivan et al., 2001), emotional (e.g., fear, anger; Thibodeau et al., 2013; Trost et al., 2012), behavioral (e.g. avoidance, endurance; Hasenbring & Verbunt, 2010; Vlaeyen & Linton, 2012) and social factors (e.g. work-related and economic factors (Haukka et al., 2011; Rios & Zautra, 2011)) that accompany the chronic pain. It is important to note that the extension code should be used only in cases in which there is positive evidence that psychosocial factors contribute to the cause, the maintenance or the exacerbation of the pain or the associated disability, or when the chronic pain results in negative psychobehavioral consequences (e.g. demoralisation, hopelessness, avoidance, withdrawal). Assigning the code requires ascertaining the psychosocial factors, e.g. by use of exploration of the patient and / or psychometric questionnaires. The inference “no somatic cause of the pain can be found, therefore the pain must have a psychological cause” is flawed and cannot form the basis of a use of the extension code “with psychosocial factors”. Assigning the code does not entail any specific causal path: the psychosocial factors can be the consequence of the burden of living with chronic pain just as much as a mechanism contributing to the experienced functional interference. The intended use of the code is communicative – the possible presence of psychosocial factors should be discussed between patient and clinician, their presence recorded and communicated to other health providers with the diagnosis. Ideally, they are used to point to a treatment relevance of the psychosocial factors. In the future, such a code should entitle the person to multimodal care including psychological treatments.

## Discussion

### Empirical Support for the New Chronic Pain Classification

The classification of chronic pain in the ICD-11 was developed with a view to the empirical evidence accrued over many years. The classification and its implementation itself have also undergone first empirical evaluations. Important targets of the revision process of the ICD were clinical utility and international applicability of the new classification (Jakob, 2018a; Reed, 2010; Üstün & Jakob, 2005; Üstün et al., 2007). Clinical utility can be regarded as an approximation of validity and reflects how much a classification system offers a useful conceptualization of the diagnostic entities, enables selecting of adequate treatments, and is easy and feasible to use. High clinical utility allows application in routine practice, facilitates communication and documentation and – ideally – is predictive of treatment outcomes. (First et al., 2004; Keeley et al., 2016)

The integrity of the diagnostic categories is an important prerequisite for the utility of a classification. Diagnostic categories should not overlap, but have clear boundaries (distinctness); together, the categories should cover the whole phenomenological space (exhaustiveness). These aspects were investigated in formative field tests (Barke et al., 2018). In a sample of unselected patients, the categories demonstrated good distinctness and exhaustiveness: less than 3% could not be assigned one of the seven main categories of chronic pain, thus dramatically reducing the number of patients who received a diagnosis reflecting a non-descript remainder category. This favorable result has since been confirmed by a documentation-based retrospective coding study (Zinboonyahgoon et al., 2021).

As a further condition, clinical utility requires reliability of the code assignments. The WHO led extensive field tests of coding aspects of the ICD-11. The results obtained for chronic pain showed that the ICD-11 diagnoses outperformed ICD-10 on all counts, including correct code assignments, ease of application, level of detail and fewer perceived ambiguities (Barke et al., 2022). A next step in reliability testing was testing the interrater-reliability of clinicians assigning diagnoses to real consecutive patients. In an international field testing study, consecutive patients were independently diagnosed by two clinicians and substantive Kappa coefficients for interrater reliabilities reported (0.596 < κ < 0.783) (Korwisi, Garrido Suarez, et al., 2022).

The clinicians were asked to rate the clinical utility of the diagnoses and it was rated as high throughout all studies (Barke et al., 2018; Barke et al., 2022; Korwisi, Garrido Suarez, et al., 2022). In addition, preliminary results of a survey among people with the lived experience of chronic pain also showed that they judged the new diagnoses to be helpful for communicating with health professionals, their families and others (Korwisi et al., 2019). The detailed categories increased the visibility of the chronic pain diagnoses when compared with ICD-10 diagnoses (Zinboonyahgoon et al., 2021).

International applicability was addressed in a multi-country field testing study in India, Cuba and New Zealand. Details of the testing are described in the study protocol (Korwisi et al., 2020). Clinicians in specialist pain centers in each country were introduced to the ICD-11 classification in training workshops and subsequently coded *n* = 353 consecutive patients with the ICD-11 classification as well as their usual diagnostic system. They provided data for the interrater-reliability and rated the clinical utility of the ICD-11 and the standardly used system, showing a clear preference for the ICD-11 classification (Korwisi, Garrido Suarez, et al., 2022). This study provides evidence that the classification is clinically useful in a range of international settings, including countries with limited resources.

### The Relationship With the Diagnoses in the Chapter on Mental and Behavioural Disorders

The ICD-10 chapter on mental and behavioural disorders includes the group of somatoform disorders, with a subdiagnosis on somatoform pain disorder. This led to several critical comments. The mind-body-dualism seemed to be amplified with this somatoform pain diagnosis, because a psychological etiology of pain conditions was emphasized in its definition. However, the whole category of somatoform disorders was associated with various problems (Creed, 2006). Despite substantial prevalence rates of 9% and above in the general population (Creed et al., 2012), in countries like the US, these diagnoses were rarely used (Dimsdale et al., 2011). Based on this critique, DSM-5 decided to revise this chapter substantially, and introduced the somatic symptom and associated disorders category. The relevance of whether somatic symptoms are medically explained or not was completely abolished, while psychological factors that are associated with the suffering from these physical complaints play a major role for the diagnosis of a somatic symptom disorder (Rief & Martin, 2014).

The ICD-11 decided to introduce a new category on “Disorders of bodily distress and bodily experience”, and its prototypic diagnosis is called “Bodily Distress Disorder (BDD)”. BDD has a similar concept to somatic symptom disorder in DSM-5: it requires bodily symptoms that are persistent, and present on most days for at least several months. As a psychological criterion, excessive attention is directed toward the symptoms. While the description acknowledges that pain symptoms are among the most common symptoms of BDD, no pain subtype is defined yet. It remains unclear whether the German modification will stick to the current F45.41 diagnosis of chronic pain with psychological and somatic factors. Therefore, at this stage, we recommend the chronic primary pain diagnoses if chronic pain is the leading somatic complaint, and the other criteria for chronic pain are fulfilled.

### Future Directions

Over the next 5-10 years, the ICD-11 will be implemented in many European countries’ health systems (World Health Organization, 2022a). Even in countries in which it is not the basis for health planning and reimbursement, governments will provide data based on ICD-11 diagnostic categories to the WHO in fulfillment of treaty obligations for the reporting of health data. It is recommended that for pain research the new diagnoses are used to inform research programs and utilize the improved diagnostic criteria as well as the specifiers (Barke et al., 2020; Treede et al., 2019). Implementing changes in classification entails changes in other areas, including adaptations in administration and information technology, reimbursement practices and student education. In addition, it requires thorough training for clinicians, administrative and coding staff. A helpful resource when beginning to familiarize oneself with the ICD-11 and the new chronic pain diagnoses, may be a paper in which questions regarding the classification were collected systematically and answers provided (Korwisi, Barke, et al., 2022).

To improve the diagnostic reliability further and facilitate the training, a classification algorithm (CAL-CP) was developed (Korwisi, Hay, et al., 2021) that guides the users through the criteria and diagnoses with a binary decision tree. The user decides for each diagnostic criterion whether it is present in a given patient and then follows the respective “yes” or “no” arrow. The decision tree guides the user through all levels that are available for the new diagnoses. In some settings, a less specific diagnosis might be sufficient (e.g., MG30.0 Chronic primary pain in primary care) while the most specific diagnoses will probably be required in pain research and specific pain treatment (e.g., MG30.02 Chronic primary musculoskeletal pain: Chronic primary low back pain). Hence, the algorithm is a central tool to apply the new diagnoses in practice as well as in research.

The clinicians participating in the international field test had used a pilot version of the algorithm and rated it favourably (Korwisi, Hay, et al., 2021). Currently an authorized version (a pdf with active hyperlinks) is available as digital supplement to the original publication (http://links.lww.com/PAIN/B277). A large test using online virtual patients is underway and its results will provide the basis for a digitized version.

A further aspect, which will have to be discussed and decided on a national level, will be the implications of the new diagnoses in terms of treatment authorization and reimbursement policies. Since the new diagnoses are based on the biopsychosocial model and it is recommended that chronic pain is no longer classified as a somatoform disorder, in some health systems, political and professional negotiations may be required to allow multimodal and interdisciplinary treatments including psychological interventions to be offered and reimbursed by multidisciplinary teams. For instance, in Germany, psychotherapists and psychosomatic hospitals are currently limited to treating disorders that are classified in the ICD-10 chapter V (Mental and Behavioural Disorders). Clinical psychologists and other health professionals working with people with chronic pain need to be aware of these developments in their respective countries and should seek to advocate for state of the art multimodal treatments for patients with chronic pain delivered by those who are qualified practitioners.
